# Purulent Meningitis as an Unusual Presentation of *Staphylococcus aureus* Endocarditis: A Case Report and Literature Review

**DOI:** 10.1155/2011/735265

**Published:** 2011-03-28

**Authors:** Giancarlo Ceccarelli, Gabriella d'Ettorre, Vincenzo Vullo

**Affiliations:** Department of Public Health and Infectious Diseases, University of Rome “Sapienza”, 00155 Rome, Italy

## Abstract

On presentation of *Staphylococcus aureus* endocarditis, unusual manifestations may represent the main clinical features of the disease. Isolated bacterial meningitis as the first manifestation of endocarditis is considered to be an unusual neurological complication. Here, we describe a case *S. aureus* endocarditis presenting as isolated meningitis and mimicking meningococcal septicaemia. Because of the high mortality rate of the disease, the prompt recognition of this infectious syndrome is of crucial importance for the correct management of patients.

## 1. Introduction

*Staphylococcus aureus *is a leading cause of bacteraemia and endocarditis, but also a rare cause of bacterial meningitis. The mortality rate of *S. aureus *endocarditis is approximately 20–40%, depending on the extreme variability in clinical presentation which may delay the early diagnosis and treatment of the disease [1–3]. Therefore, early diagnosis and adequate monitoring of the various complications due to *S. aureus *endocarditis are important. In this paper, we describe a case of *S. aureus *endocarditis presenting as isolated meningitis and mimicking meningococcal septicaemia.

## 2. Case Report

A 40-year-old male was hospitalized following 5 days of fever, lethargy, and mental confusion. He had been taking antipyretics and Amoxicillin + Clavulanic acid 1 g 2 times/daily as prescribed by a physician for 5 days without any benefits. From his medical history, there was no evidence of previous neurological, respiratory, cardiovascular, gastrointestinal, and renal disorders. On admission, the patient was lethargic and mentally confused. His vital signs were as follows: body temperature 39.4°C, blood pressure 100/60 mmHg, heart rate 112/min, and respiration rate 32/min. Physical examination revealed photophobia and nuchal rigidity. Kernig's sign and Burdizinski's signs were both positive. There were no abnormal findings in the cardiovascular, respiratory, and gastrointestinal system. Laboratory studies were as follows: white blood cells (WBC) 23.4 × 103/mmc with 93% neutrophils and 3% lymphocytes; red blood cells (RBC) 4.2 × 106/mmc; haemoglobin (Hb) 11.6 g/dL. There were no alterations of renal and hepatic function; electrolytes and glycaemia were within normal values; erythrocyte sedimentation rate was 82; C-reactive protein 7 mg/dL. Coagulation data demonstrated INR 1.29 ratio, fibrinogen: 964 mg/dL; fibrin split products: 1169 *μ*g/mL. Chest X-ray did not show alteration. ECG examination showed typical findings of tachycardia with a regular rhythm. Computed tomography (CT) scan of the brain was negative for space-occupying lesions and intracranial hypertension. 30 minutes after admission into hospital a lumbar puncture was performed and cerebrospinal fluid (CSF) findings showed opalescent CSF with WBC 962/mmc (90% neutrophils and 10% lymphocytes), glucose 70 g/dL, protein 105 g/dL, and CSF Gram stain smears negative. Within 4 hours from admission, there was evidence of septic shock with diffuse pustular purpuric lesions. Based on the CSF findings as well as systemic signs, a presumptive diagnosis of meningococcal sepsis was made and ceftriaxone (2 g every 12 hours I.V.) was administered. 48 hours after admission, the patient still had fever and WBC were 19.4 × 103/mmc with 87% neutrophils. After 3 days from admission, physical examination revealed abdominal tenderness and pain, and showed a 2-3/6 systolic ejection murmur at the left sternal border proceeding to neck blood vessels. Therefore, transthoracic echocardiography was performed; it revealed a vegetation adherent to the posterior leaflet of the mitral valve with mitral regurgitation consistent with mitral valve endocarditis; these findings were confirmed by transesophageal echocardiography.

A following total body CT scan showed multiple splenic spots compatible with abscesses and one small abscess on left psoas muscle. Moreover a methicillin sensitive *S. aureus *(MSSA) was isolated in two blood cultures collected on the first day from admission, while the CSF cultures were negative. Based on these findings, on the third day of admission, antibiotic therapy was changed to oxacillin (2 g I.V. every 4 hours) plus gentamicin (1 mg/kg I.V. every 8 hours). One week after the beginning of the new therapy, the patient showed a progressive defervescence, reduction of abdominal pain and skin lesions, nuchal rigidity and lethargy slowly resolved, the leukocyte count had reduced considerably, and all blood cultures were negative. The transthoracic echocardiogram showed a reduction of the vegetation on the mitral valve. A total body CT scan was repeated after 10 days of treatment and it excluded the presence of embolism; it also showed a reduction of the splenic and psoas muscle abscesses. After 6 weeks of therapy with Oxacillin and Gentamicin, the patient was free of symptoms and blood plus instrumental examinations showed absence of the previous findings. The patient later had a checkup involving full blood examinations, transthoracic and transesophageal echocardiography, 1 month and 3 months after discharge from hospital, these all showed absence of disease and a total body CT scan done at the end of the 3 months followup was also negative; the patient was therefore considered disease-free and was committed to a cardiologist for monitoring mitral regurgitation and heart function.

## 3. Discussion

Serious *S. aureus *infections caused by both resistant and susceptible strains are increasingly reported in community and hospital settings [4]. In particular, infective endocarditis represents the most serious *S. aureus *bacteraemia complication. *S. aureus *is a unique pathogen because of its ability to cause endocarditis often involving previously architecturally normal cardiac valves. *S. aureus* endocarditis also can have profound and devastating neurologic consequences. The frequency of neurological complications due to endocarditis was found to remain constant despite therapeutic advances and profound epidemiological changes [5]. Jorge et al. [6] studied a 222 patients cohort with infective endocarditis: 17 patients presented meningitis as unique manifestation of neurological complication associated to the diagnosis of infective endocarditis, 56 patients presented neurological complication, and the others were without any neurological complication. Also Brouwer et al. reported that 89% of patients with community-acquired *S. aureus *meningitis at the admission in the hospital had foci of infection outside the central nervous system [7]. The incidence of neurologic complications of infective endocarditis is dependent on the organism and valvular location; the highest incidence is 87% with *S. aureus* vegetations on the mitral valve. Jorge et al. described that patients with concomitant *S. aureus *meningitis and endocarditis had a higher mortality (56%) related to age, shock, and infection with phage type 95 strains [6]. Fong and Ranalli reported that meningitis associated with endocarditis caused by *S. aureus *showed a more fulminant course [8]. Comparative analysis showed a greater mortality rate in presence of neurological complications and meningitis, and *S. aureus *was the predominant etiological agent [9]. 

The risk of occurrence of stroke, hemorrhage in CNS, purulent meningitis, and brain abscess exists in patients with infective endocarditis; anyway their clinical manifestations are often indistinguishable from those present in the same diseases in the absence of endocarditis. Embolic events or autoimmunity may underlie the etiology of stroke but the cause of CNS hemorrhage in infective endocarditis is not completely clear and nearly 30% of hemorrhagic cases are due to staphylococci. Also nonfocal symptoms or encephalopathy can be the result of microscopic emboli and subsequent ischemia. Pathological findings evidence that macroscopic brain abscesses are rare but microscopic abscesses are more common and often are discovered at autopsy. Due to the fact that clinical manifestations are often indistinguishable from those present in the same diseases in the absence of endocarditis, it is useful to consider the possibility of concomitant infection of a heart valve in case of presence of fever, alteration of inflammatory markers, and/or *S. aureus *bacteremia. Anyway mental status changes, amnesia, nonfocal symptoms, transient ischemic attack-like, and fluctuating focal neurologic signs can herald a diagnosis of infective endocarditis [10–14].

Neuropsychiatric symptoms may be the first manifestation of an underlying cardiac disease. A study by Bademosi et al. indicated that 38% of patients with infective endocarditis had neuropsychiatric manifestations and this may also occur in *S. aureus *endocarditis [15]. Laboratory and imaging studies of neurologic consequences of endocarditis include cerebrospinal fluid examination, CT of the brain, and magnetic resonance imaging (MRI) that can reveal focal areas of ischemia even in the absence of clinical manifestations of a CNS disorder. Patients with community-acquired *S. aureus *meningitis have a WBC count and CSF protein levels usually lower than those with other pyogenic meningitis, and the CSF Gram stain is positive in only 40% of cases [1–3]. Anyway Røoder et al. described that only <2% of patients with *S. aureus *endocarditis developed acute bacterial meningitis with positive CSF cultures [10]. The isolation is more probable from blood cultures than from the CSF [16, 17]. In our case paper, the CSF cultures were negative but the patient had an antibiotic treatment before admission and lumbar puncture. However, in cases of community-acquired bacterial meningitis, any atypical isolate such as *S. aureus *should be considered suspicious for the presence of another localization of the infection [18, 19]. 

Spontaneous *S. aureus *meningitis as a consequence of endocarditis may pass unnoticed initially, because a murmur is absent at presentation in 30% of cases [3]. In our patient, murmur was found during the third day of hospitalization; moreover, skin lesions observed in meningococcal sepsis and in *S. aureus *sepsis are similar. For that reason, it is important to search for an underlying cardiac cause in patients who present with meningitis and skin lesions in a clinical course that may mimic the classical presentation of meningococcal sepsis. The presence of pustular skin lesions in a patient with sepsis could be suggestive of *S. aureus *as the pathogenic agent. In fact, in staphylococcal endocarditis purpuric lesions may progress into cutaneous gangrene or may be pustular, with organisms present on Gram stain [20]. However, sometimes other organisms (such as *Streptococcus pyogenes*, *Streptococcus viridians*, and *Pseudomonas aeruginosa*) may be involved in the event of an occasional pustular rush. Morover, although the gonococcal endocarditis is now rare, also *Neisseria gonorrhoeae* should be considered a possible etiologic agent of endocarditis in the presence of pustular lesions [21]. Microbiologic examination of samples of secretions and biopsies of the petechial rash, purpura, and pustules may be useful for revealing the causative agent. Another two well-known skin manifestations of bacterial endocarditis are Janeway lesions and Osler nodes. Janeway lesions are more commonly seen in acute endocarditis, when bacteria (most often highly virulent organisms such as *S. aureus*) may be cultured from them and the histology is usually consistent with septic microembolism [22–25]. 

Distinguishing patients with uncomplicated *S. aureus *bacteremia from those with endocarditis is therapeutically and prognostically required, but often difficult. The clinical examination and the traditional clinical predictors of endocarditis (as community acquisition, absence of an obvious primary focus and evidence of metastatic foci) in patients with *S. aureus* bacteremia often fail in establishing a diagnosis of endocarditis [26]. Therefore, echocardiographic evaluation should be considered part of the early evaluation of patients with *S. aureus *bacteremia [27, 28]. For these reasons, in the last clinical practice guidelines for the treatment of methicillin-resistant *S. aureus* infections, echocardiography is recommended for all adult patients with bacteremia and transesophageal echocardiography is preferred over transthoracic echocardiography [29]. 

In our case, we carried out serial echocardiographic examinations in order to monitor the clinical course of the disease; in fact *S. aureus* can be a cause of destructive valve endocarditis, and its pathogenic potency is explained by the expression of binding factors promoting the adherence to valves as fibrinogen binding protein [30]. Moreover, we performed both transthoracic echocardiography and transesophageal echocardiography; this is because transesophageal echocardiography is a highly valuable adjunct to other diagnostic tests and would appear to be of benefit in patients with a risk of complicated clinical course who may have additional cardiac lesions not demonstrated by transthoracic echocardiography [31, 32]. In fact, transesophageal exam may be advisable to evaluate perivalvular extension of disease, unsuspected paravalvular abscess, vegetation size, and other factors which may inform surgical decision-making [33]. *Staphylococcus aureus* infection is associated with a higher recurrence of embolization, and septic embolization has important effect on long-term survival resulting in death in 24% to 50% of those who sustain an embolus [34]. In addition, a significant proportion of patients with endocarditis may also have clinically occult embolic locations: for these reasons we performed serial CT scan examinations in order to monitor the clinical course of embolic complications from endocarditis. Moreover, recently also PET/CT scan has been proposed as an important diagnostic tool for tracing peripheral embolism and metastatic infection in the acute setting of infective endocarditis, since a PET/CT scan detected a clinically occult focus in nearly one third of episodes [35]. In our case the presence of splenic abscess was an important risk factor for rupture of the spleen, because splenic tissue is very fragile (especially if the abscess is located subcapsularly), and a splenic rupture can result from minimal trauma [36]. In these cases, a successful outcome lies with a choice between medical and surgical treatments (splenectomy); however, there is still insufficient evidence in the decision-making process [37, 38]. 

In summary, *S. aureus *endocarditis carries a mortality rate of 20–40%: it depends in part on the extreme variability in clinical presentation, which may delay the early diagnosis and treatment of the disease. In case of bacterial meningitis associated to infective endocarditis, the mortality rate approaches to 60% [9]. Isolated bacterial meningitis as the first manifestation of endocarditis is unusual notwithstanding neurological complication [2]. The association of neurological symptoms with skin lesions may mimic the presentation of meningococcal meningitis with septicaemia in absence of classical cardiac signs of *S. aureus *endocarditis [3]. 

In this paper, we emphasize the importance of searching an underlying focus of *S. aureus *endocarditis in a patient presenting as purulent meningitis and with a presumptive diagnosis of meningococcal sepsis, even in the absence of typical symptoms and/or signs of endocarditis. Because of the high mortality rate of the disease, the prompt recognition of this infectious syndrome is of crucial importance for the correct management of patients.
